# Emotional intelligence, leadership, and work teams: A hybrid literature review

**DOI:** 10.1016/j.heliyon.2023.e20356

**Published:** 2023-09-20

**Authors:** Isabel Coronado-Maldonado, María-Dolores Benítez-Márquez

**Affiliations:** a Department of Economy and Business Administration, Faculty of Economics and Business, University of Malaga, Malaga, Spain; b Department of Applied Economics (Statistics and Econometrics), Faculty of Economics and Business, University of Malaga, Malaga, Spain

**Keywords:** Leadership, Emotional intelligence, Work team, Group, Organization, Work climate

## Abstract

Emotional intelligence (EI) has been widely researched in different fields of knowledge. This paper reviews the literature on emotional intelligence, leadership, and teams in 104 peer-reviewed articles and reviews provided by the Web of Science and Scopus databases from 1998 to 2022. It is a hybrid or mixed review as it uses both quantitative and qualitative analysis techniques. The aims of this study are a performance analysis of the selected documents (years of publication, country, sectors, techniques used, most cited authors, authors with more publications, journals, journal quartiles, and scope of publication), as well as a co-word analysis using Atlas. ti v8. The results of the quantitative analysis indicate that the majority are empirical works. The qualitative analysis is a co-word analysis providing the following results: (i) classification of authors by major themes-categories (EI, leadership, team), (ii) classification of themes within each major theme: three subcategories in EI, 17 subcategories in leadership, and 19 subcategories in team and, lastly, (iii) classification according to the chronological development of main objectives from the most cited authors' articles we analyzed. Leadership (transformational, emergence, virtual, effective, health, effectiveness) is the major theme we studied. Our in-depth review of the articles has shown that emotionally intelligent leaders improve both behaviors and business results and have an impact on work team performance. It also highlighted a positive relationship between emotional competence and team members’ attitudes about work. The new trends focus on the impacts of COVID19, the global crisis due to the Ukraine War, working in VUCA and BANI environments, comparative studies between generations, the application of artificial intelligence and the influence of mindfulness on organizations.

## 1 Introduction

Emotional intelligence (EI) involves understanding others in a social context in such a way that it enables one to detect nuances in emotional reactions and use this knowledge to influence others by controlling and regulating emotions [1]. It is therefore a crucial element of the competencies that are necessary for effective leadership and teamwork performance.

Although the intelligence quotient is considered to be one of the most significant predictors of future success in life, the need for effective leadership has led researchers and scholars to seek other improvement tools. Studies such as those conducted by Goleman [2] on competency assessments show that emotional competencies account for two out of three essential skills for effective performance in a wide array of different job positions in companies around the world. Moreover, Dulewicz and Higgs [3] point out that both academics and senior management at companies are increasingly recognizing the importance of EI in effective leadership, making it necessary to address the role of EI when discussing leadership [4].

Other researchers have suggested that not only is EI essential for an individual's success within an organization but that it becomes increasingly important as said individual moves up the ranks into leadership positions [2,[5], [6], [7]]. Various work streams suggest that EI is relevant for effective leadership in 21st-century organizations.

Organizational roles have changed over the years. Organizations now emphasize the need for leaders to take on new roles, including coordinating and facilitating others’ behavior in the workplace. Organizations therefore need to achieve and retain a sustainable competitive advantage in order to continue to develop, paying special attention to human issues [[8], [9], [10], [11], [12]]. Furthermore, many employees have to work in teams in order to achieve complex organizational objectives and work groups are becoming more common in organizations [13]. It is clear that EI influences relationships within the team [14].

Some authors highlight the importance of emotional intelligence in leadership and work teams [15]. According to Lim et al. [16], literature reviews (as independent studies) provide new researchers with broader knowledge of a certain topic. Furthermore, reviews that include both conceptual and empirical studies lend them greater credibility, as well as helping to suggest important gaps in the literature and highlighting contradictory findings. The justification for this semi-systematic review [17] of emotional intelligence, leadership (in the broadest sense of the word), and work groups is that our performance and co-word analysis is more current and comprehensive, including both empirical and conceptual studies.

Furthermore, taking into account current reviews on emotional intelligence and human resources, we can cite the systematic review by Sharma and Tiwari [18]. Our research complements this study for several reasons. First, these scholars used Web of Science (WoS) and Google Scholar for their review while our review considers WoS and Scopus, which includes peer review scholar journals as Google Scholar does not index with any quality assessment. Secondly, the abovementioned authors selected literature from the last two decades (2002–2022), while our analysis is not limited to any specific years of publication, thus offering a more historical and simultaneously current view. Third, the other authors focused their attention on transformational leadership while our study encompasses all kinds of leadership. Fourth, our study includes both empirical and conceptual work while the former authors included only articles. Lastly, the other authors focused on personnel performance, which is further complemented by our approach to work groups, as we also consider other issues such as stress, harassment and worker burn-out.

This paper presents a literature review on EI, leadership, and work teams in peer-reviewed articles written in English and is divided into five sections. This introduction is followed by Section 2, which provides details regarding definitions and assessments of EI and explores how EI relates to leadership and teams in the leading research. Section 3 describes the methodology used to conduct the review including the search strategy, document selection, and analytical techniques applied (performance analysis and co-word analysis) related to these three topics aforementioned. Section 4 shows the results for the 104 selected articles, including a chronological development of the main articles’ objectives and findings from the most cited authors. Section 5 presents conclusions and future lines of research.

At this point, we have established the following research questions.

RQ1 Do all the articles from the search strategy go as in-depth into the three topics under review: EI, leadership and work teams?

In regard to performance analysis, we have considered the following research questions.

RQ2 What evolution can be seen in the articles published per year from 1998 to 2022?

RQ3 Which countries samples have been researched?

RQ4 Which sectors are studied in the analyzed articles?

RQ5 Who are the most cited authors?

RQ6 Which authors have the most published articles? What is the ranking of the scientific journals that have published the analyzed articles?

RQ7 What is the classification of documents by type of research design, distinguishing between conceptual and empirical, and how many are qualitative and quantitative inside? What techniques are used in the empirical quantitative and quantitative studies?

RQ8 What EI assessment measures or models are applied in the analyzed documents?

Regarding co-word analysis, we propose the following questions.

RQ9 Which are the main categories resulting from the co-word analysis and the authors publish in each one?

RQ10 Which themes are studied in the subcategories resulting from the co-word analysis and who are the authors publish in each one?

RQ11 Which research objectives have been studied and their correspondent authors?

## 2 Theoretical background

### 2.1 Emotional intelligence

The term EI is rooted in the term “social intelligence.” While Thorndike [19] defined EI as “the ability to understand people,” Gardner [20] uses this term in his multiple intelligence theory to refer to interpersonal and intrapersonal intelligence. According to Ruisel [1], EI is a type of social intelligence that is widely recognized as important in most cases. Wechsler's [21] work encouraged the study of non-cognitive intelligence, including IE, suggesting that the totality of intelligence should necessarily include a measure of cognitive and non-intellectual effects [22].

Salovey and Mayer [23] introduced the specific definition of EI and then later simplified the definition as “the ability to perceive and express emotion, assimilate emotion and thought, understand and reason with emotion, and regulate emotion in the self and others” [24] cited in McCleskey [25].

Although Goleman [26] may be regarded by some as the father of the concept of EI due to his highly acclaimed book chapter, “The Emotional and Intelligent Workplace,” it is actually an adaptation from Salovey and Mayer [23], Mayer and Salovey [24], and many other researchers who had previously conducted studies in this field. In 1997, Goleman stated that we have to manage our emotions without letting them overwhelm us, motivate ourselves to do work, to be creative, and to feel what others feel while effectively managing our emotions. Goleman [2] defines EI as “the ability to recognize our feelings and those of others, to motivate ourselves, and to handle our emotions well to have the best for ourselves and our relationships.” It is also relevant to mention the very concise definition by Martinez [27] as “an array of non-cognitive skills, capabilities, and competencies that influence a person's ability to cope with environmental demands and pressures.”

Following Mayer et al. [28], other researchers such as Barn-On [29], Goleman [2], and his colleagues complemented this concept with different types of personality traits and skills. Barn-On [29] states that EI is a set of non-cognitive abilities, competencies, and skills that influence a person's ability to successfully cope with the demands and pressures of the environment. Other studies consider EI to be a personality trait [[30], [31], [32]]. The latter academics define it as “a constellation of emotional self-perceptions located in the lower levels of personality hierarchies, measured via the trait emotional intelligence questionnaire.”

Other scientific studies that are worthy of mention bring up concepts such as emotional literacy [33,34]; personal literacy [35]; interpersonal intelligence [20,36], and socio-emotional competencies [37]. So, following Goleman's [2,38] definition, Boyatzis [39] considers EI in terms of competencies and skills, defining an EI competence as the ability to recognize one's own feelings as well as others' feelings. There are three such competencies: cognitive competencies, social intelligence competencies, and EI competencies.

After reviewing the theoretical perspective of EI, early studies attempted to relate EI to various variables that are not directly observed (including factor, composite, and latent variables or constructs, among others) in order to assess an individual's capacity for EI. Researchers consequently began to develop several EI models and scales [23,29,32]. Current scales generally achieve appropriate psychometric properties, although some have limitations.

Generally speaking, three different approaches have been used to evaluate EI. Mixed or trait models measure an individual's EI through questionnaires and self-reports, regarding EI as a personality trait that includes social, emotional, and organizational aspects or competencies. In contrast, ability models regard EI as the result of the ability to solve certain emotional issues, comparing these responses with pre-established scoring guidelines according to specific measures [40,41]. The third approach is external assessment, which involves asking someone other than the test-taker to give their opinion and assessment of how they perceive the person being evaluated. This technique involves evaluating interpersonal skills, which compliments and reaffirms the two former techniques and avoids any issues related to social desirability presented by certain individuals.

In regard to self-report measures, the theoretical conceptualization of Salovey and Mayer [23] led to the classic version of the “Trait Meta-Mood Scale-48” (TMMS-48). Later, Schutte et al. [32] produced the Schutte Self Report Inventory (SSRI) measure. Similarly, Wong and Law [42] eventually developed Wong and Law's Emotional Intelligence Scale (WLEIS) to assess perceived EI.

Other self-report measures relate to emotional, social, and occupational functions. Bar-On [29] developed the Bar-On Emotional Quotient Inventory (EQ-i) measure, which includes items intended to ensure that participants do not distort the possible consequences of social desirability [43]. Petrides and Furnham's [44] developed a measure based on the Barn-On [29] scale, defining EI as a set of personality traits, socio-emotional competencies, motivational aspects, and cognitive skills. They created the Trait Emotional Intelligence Questionnaire (TEIQue). Similarly, Goleman's base model [2], the “Emotional Competence Inventory” (ECI), initially included a series of emotional competencies. Boyatzis et al. [45] eventually reduced the dimensions of the aforementioned assessment so as to only include business-related competencies of workers and leaders. All of these versions generally have acceptable levels of consistency and, according to Extremera et al. [46], most of them have reduced versions of the measures from the original model.

Task and performance-based ability and performance measures have been developed in order to mitigate potential biases that may appear in self-reports, as one may feel that perceived EI is associated with high social desirability. The most widely used performance measure is the Mayer-Caruso-Salovey [28] Multifactor Emotional Intelligence Scale (MEIS) based on theoretical concepts developed by Mayer and Salovey [24], which generally offers a high degree of predictive ability. Mayer et al. [28] eventually proposed another version of the theoretical model, the Mayer-Salovey-Caruso Emotional Intelligence Test (MSCEIT), which no longer includes scales with low reliability (as it did in the two former versions, v.1.1 and v.1.2).

There are various EI-related controversies. Murphy [47] and Mayer et al. [48] argue that many different constructs lead to suspicions about whether or not they are interrelated, which is detrimental to the legitimacy of the term emotional intelligence. Locke [49] criticizes the great diversity of shifting and incomprehensible definitions of EI and proposes replacing the term EI with introspective ability. In the same vein, Cherniss [50] and Conté [51] criticize the fact that existing assessment methods measure intelligence based on different conceptualizations of the term and that there's a lack of consensus among them. Conté [51] discusses whether these concepts are even comparable.

Generally, these scales have a high degree of reliability and internal consistency; however, the scales do not indicate whether they assess concepts that are already measured by other more established constructs. Mayer et al. [28] and Brackett and Mayer [52] have concluded that there is a low correlation between the MSCEIT model and the EQ-i model constructs. They raise the question of whether these models measure the same concept [53]. Rosete and Ciarrochi [54] found no significant correlations between the total score of the EI construct and any of the 16 personality factors in the ability-based MSCEIT model. Wilhelm [55] argues that, despite possible discordances, the MEIS and MSCEIT are the most comprehensive assessments of EI. He considers the MSCEIT to be the most ambitious and appropriate approach to date for the overall assessment of emotion-related abilities. Wilhelm [55] also recommended not conducting any further research on self-reports. According to Mayer et al. [48] regarding the Big Five measures, the strongest correlations between EI and the five personality dimensions only occur with the agreeableness factor. Furthermore, MEIS and MSCEIT (v.2) measures seem to correlate better with general mental ability than with self-report measures [51,56]. In contrast, proponents of EI measurement find it acceptable that the different measures have adequate internal consistency reliability, both in self-reports and ability reports [2,29,30].

### 2.2 Emotional intelligence and leadership

Since its conceptualization in the early 1990s, EI has been considered as a relevant non-observable variable associated with organizational success. It is important to note how we manifest and control emotions. Several authors [2,23,29,57] share multiple theoretical foundations on this issue, including: awareness of our own emotions, awareness of others’ emotions, and our understanding and ability to manage our emotions and those of others.

The importance of emotions in the workplace has led researchers to increasingly recognize that effective leadership can also have a strong emotional component, making it vital for leaders to be emotionally intelligent [38]. Consequently, effective leadership may depend heavily on a leader's ability to both proactively and reactively manage the emotions of those under them [58,59].

According to studies from the last few decades, it is important to study personalities and individual differences in order for leadership to be effective [[60], [61], [62]]. These studies have led to the emergence of the EI perspective, which is the ability to recognize, understand and manage one's and others' moods and emotions, as established by Salovey and Mayer [23]. Likewise, Chen et al. [63] state that EI has an impact on success in leadership positions.

Although most studies on workplace leadership address constructive forms of leadership, another aspect that has been considered recently is the importance of toxic and counterproductive work behaviors that can occur in organizations [64]. Most studies focus on employees in lower-level positions [65], while few have touched on issues that may expose people in managerial, supervisory, or leadership positions.

Supervisors' abusive behavior towards their subordinates has an adverse effect on employees’ work behavior and performance, including decreased job satisfaction and commitment to the organization [66]. Consequently, employees feel helpless and more conflicts arise regarding their roles and employee turnover [67].

The “Five Factor Model” personality studies conducted in recent years encompass personality traits such as extroversion, empathy, conscientiousness, and neuroticism. Multiple studies explore the influence of these general factors on personality dimensions, performance, and leadership at work [68,69].

### 2.3 Emotional intelligence and work teams

On an individual level, team members’ personalities, abilities, and skills play an integral role in the work team. Highly emotionally intelligent individuals can communicate effectively and empathize with others, allowing them to develop cohesive, supportive relationships [70,71]. Furthermore, emotionally intelligent individuals can think in an innovative manner and create an environment that supports these activities [72]. Accordingly, some studies show that EI competencies are significantly related to individual performance [73].

Because business environments experience continuous changes, leadership positions often require more than just competence to perform tasks or technical expertise [74]. Scholars have explicitly demonstrated that emotional skills are essential to a leader's performance at the executive level [15] and are becoming increasingly important as individuals move up in their organizational hierarchies [3,75]. Consequently, EI is a crucial element in the workplace, and companies and individuals are growing increasingly interested in balancing the rational and emotional aspects of business strategies [76]. As social and individual skills improve, so does the ability to express inner feelings toward others; EI is therefore manifested in one's effectiveness on the job [77].

Druskat and Kayes [78] suggest that a group's ability to manage itself both on a group and individual level plays a crucial role in developing social relationships, effective task processes, and overall group effectiveness. Druskat [79] and, later, Druskat and Wolff [80] proposed a model of group EI that analyzes the role of emotional disturbances in work teams, further developing and recognizing the existence of these enforced emotionally intelligent group norms. Druskat and Wheeler [81] link the degree to which a group develops these norms to team performance, considering group EI to be a “set of group norms that shapes group members' interpretation of emotion-provoking events and group members' response to that interpretation.”

As leaders have a direct influence on their employees, it is easy to see how a team leader's EI can influence the development of emotionally competent group norms in his or her team [82]. Consequently, a team leader's EI strongly impacts the work team's well-being as the work climate becomes stressed [83]. Furthermore, emotionally competent leaders perform better and are more successful [2,74,84]. The idea is to link these emotional competencies with leadership behaviors and organizational performance [74,[85], [86], [87]].

Since teamwork is an intrinsically social activity, emotions play an essential role in team effectiveness and affect team behavioral outcomes [88]. EI is therefore essential for effective team interaction and productivity. In parallel, recognizing and managing emotions is crucial for both individuals and work teams [2,23,34,38,57,75,89].

### 2.4 Current research trends

Ongoing business challenges are affecting workers for a variety of reasons, including changing organizational structures, adoption of new technologies, and working remotely. These changes are leading to major transformations in how organizations work as well as the actual space at the workplace, both of which are influenced by external factors and new trends.

One of these trends has arisen from the global crisis caused by the COVID-19 pandemic, which made it especially important to know how to manage fear and stress. This situation did not promote emotional or physical well-being in people and this imbalance was transferred to both the personal and the professional levels for all workers within an organization. The changes that have occurred in managing the workforce remain in the post-pandemic era and, therefore, must continue to be assessed, especially considering that the markets are still volatile, uncertain, complex, and ambiguous (VUCA). Likewise, the same goes for the changes brought about by the war in Ukraine, not only on an economic level, but on a personal one too [90]. In wartime conditions, ensuring economic and financial security at all social and economic levels must be considered a priority. For post-war recovery of companies with critical infrastructures in global BANI conditions, developing a new prototype of strategic management for financial and economic security will make it possible to expand the limits for applying effective security-oriented management tools. All the uncertainty caused by the uncertain future itself has a negative impact on levels of productivity, loyalty and the manifestation of leadership qualities [91].

In today's brittle, anxious, non-linear and incomprehensible (BANI) environment, emotional intelligence, leadership and work teams have changed significantly, presenting new strategies to cope with this environment. In this context, Sharma and Tiwari [18] point out that individuals who have a high level of emotional intelligence are better able to manage pressures and demands at work because they are more self-aware, according to studies by Fisher et al. [92], Hartmann et al. [93], and Wang et al. [94]. This means that BANI environments can be coped with by creating a culture of resilience in order to have rapid recovery processes in very uncertain situations. Resilience can be a skill that can be developed or learned, not only in order to cope with problems but also as a means of learning and improving workers' success rates [95]. Considered from this perspective, emotions are an integral part of human beings and therefore it is important to take into consideration people with high levels of emotional intelligence. Furthermore, these resilient skills provide individuals with psychological/emotional stability, allowing them to calmly deal with stressful situations and make effective decisions [96]. Likewise, emotional intelligence serves as a prerequisite for becoming more resilient, providing a specific pathway to career success [95].

Developing and encouraging workers' skills such as empathy, confidence, intuition, creativity and self-control is another way to increase people's ability to adapt to rapidly changing and unexpected situations. Consequently, the practice of mindfulness is positively related to the development of changes in personal and social awareness as it regulates people's emotions and behaviors. Mindfulness also has a positive influence on work engagement and performance, as well as interpersonal relationships since it increases workers' personal well-being [97]. Mindfulness also improves cognitive function, contributing to the development of emotional intelligence competencies associated with higher performance and effective leadership [98]. Similarly, the use of mindfulness at work is useful for leaders to develop emotional intelligence, social skills, and support systems within the organization [99].

Another current topic is studying the behavior of different generations (Baby Boomers, Generation X, Generation Y or Millennials, and Generation Z). Specifically, in the field of employment, there are differences between these generations. Baby boomers are likely in the process of retiring, while Generation Y may have greater project management responsibilities and Generation Z is just starting to enter the workforce. The latter generation is characterized by having short attention spans, socializing on the Internet, being impatient, innovative and creative, and they like to work individually. Generation Z is associated with being born with technology and having a good command of it, unlike Millennials who started using it later on in life [100]. Magano et al. [100] have associated Generation Z with project management competencies that have to do with strengths in emotional intelligence. Significant gaps were found in traits such as individualism; that is, less personal relationships. Some studies support the importance of sociocultural factors that influence the acceptance of technology by this specific generation, although other studies also add that additional factors such as cross-cultural, religions and regions must be taken into account [101].

Another trend is the major revolution that comes with advances in technology. In order for service companies to increase their operational efficiency, artificial intelligence is being increasingly used to improve the quality of services and customer satisfaction, such as automation techniques used by managers. In particular, techniques that use machine learning models to generate content similarly to how the human brain learns and responds to data, information and indications (generative artificial intelligence) [102]. This is the case with ChatGPT and DALL-E, which shocked us with its ability to understand complex and varied human languages, creating rich and structured responses that are very similar to how a human would respond [90,103]. Nevertheless, artificial intelligence continues to be more useful for technical and operational efficiency than for people-related services, which require an understanding of human behaviors, improving employee work and managing emotional tasks [18].

## 3 Methodology

This review would be considered semi-systematic according to Snyder [17]. Two bibliometric analysis techniques were used: a quantitative technique (performance analysis) and a qualitative technique (co-word analysis), making it a hybrid review [16]. Ordanini et al. [104] suggested choosing research with a significant impact in the field such as Web of Science, and Scopus databases as these databases includes peer review scholar journals.

The search strategy used to select articles employs the advanced search TOPIC option with the words ‘emotional intelligence,’ ‘leader*’ and ‘team’, that is, TS = (leader* AND emotional intelligence AND team). Based on the WoS core collection database, we applied the following filters: document type (articles, review articles), up to the year 2022, and language (English). The search in Scopus included the article title, abstract, and keywords with the same terms (TITLE-ABS-KEY (emotional intelligence AND leader* AND team). We then exported the complete records to Excel and merged both files, eliminating duplicates. Early access documents were not considered although they are involved in trends and other sections such as the conclusions. It is important to clarify that there may be articles of leadership in any category, not restricted only business.

Applying the search resulted in a total of 564 articles and 76 duplicates were eliminated, resulting in 488 articles. At that point we could answer the first research question.

RQ1 Do all the articles resulting from the search strategy go as in-depth into the three topics under review: EI, leadership and work teams?

A manual examination by each of the authors showed that 384 articles either only made occasional references to one of the main topics (EI, leadership and team) or strayed far from the context of business organizations. Most of the studies from the final selection, 104 documents, were obtained from the WoS database, which accounted for 74 articles, or over 70% of the total publications selected and the remaining 30 articles came from the Scopus database. An asterisk (*) marks the 104 reviewed documents in the Reference Section.

The performance analysis (quantitative) provides a descriptive data analysis concerning each response of research questions from RQ2 to RQ9 displayed in the Section of results. We also mixed the performance analysis with the thematic review (qualitative) based on co-word The final selection of 104 documents was then analyzed by Atlas. ti version 8 software, which provides rigor to finding main categories, which are EI, leadership and team in the case of this study, and subcategories. The purpose of the thematic review is to find the most important research categories or themes.

## 4 Results

### 4.1 Results of the performance analysis

RQ2 What evolution can be seen in the articles published per year from 1998 to 2022?

The year of publication of the selected articles ranged from 1998 to 2022. This topic began to emerge in the literature in 1998, which makes it a relatively recent topic of interest. Interest grew in 2002, 2008, 2012, and 2020 (6%). The highest number of relevant publications were in 2017, 2018 (8%), and 2022 (7%); while the years with the lowest number of publications were prior to 2002.

RQ3 Which countries samples have been researched?

The first descriptive data in this study is categorization by sample country (Table 1). The majority of the sample articles are from the United States (24%), the United Kingdom (15%), China (13%), and Australia (8%). The remaining countries include a group of eight countries with less than 5% of the total, and the rest represent a small percentage of the sample. Also, less than 2%, one paper is from a 13 sample countries and ten papers are from more than one sample country.

RQ4 Which sectors are studied in the analyzed articles?

The most noteworthy sectors (Table 1) we considered include: Educational training (22%), Hospitals & Healthcare (17%), and Construction (12%).

RQ5 Who are the most cited authors?

The most frequently cited authors in both WoS and Scopus are: Schutte et al. [32], with 1796 citations, and Judge et al. [60], with 1396 citations. Carmeli [15] is also worthy of mention, with 407 citations in Scopus, although he is not mentioned in WoS (Table 2).

RQ6 What is the ranking of the scientific journals that have published the analyzed articles?

The analyzed articles were published in 73 different journals (Table 2). The most significant number of publications corresponds to the Leadership and Organization Development Journal (10%), followed by the Journal of Managerial Psychology (5%) and Leadership Quarterly (each with 5%). The classified categories of the Journal Citation Report (JCR) in Social Science Citation Index (SSCI) are: Business, Nursing, Industrial Relations & Labor, Management, Multidisciplinary, Psychology, Psychology, Applied Psychology, Social Psychology; and the most frequent category among them is Management, followed by Applied Psychology. Related to the ranking of top thirteen journals with more than one published paper, the ranking have been abbreviated JCR-SCCI-Quartile (Q) number and are the following: JCR–SSCI–Q1 Nursing; JCR–SSCI–Q1 Industrial Relations & Labor; JCR–SSCI–Q3 Management; JCR–SSCI–Q3 Business; JCR–SSCI–Q1 Psychology; JCR–SSCI–Q4; Applied Psychology; JCR–SSCI–Q3 Social Psychology, and JCR–SSCI–Q1 Multidisciplinary.

RQ7 What is the classification of documents by type of research design, distinguishing between conceptual and empirical, and how many are qualitative and quantitative inside? What techniques are used in the empirical quantitative and quantitative studies?

Regarding research methods, 79% are empirical and 21% are conceptual (Table 3). Out of the empirical, from 82 only two articles are qualitative and the rest are quantitative. Also, out of the quantitative, correlation analysis and regression analysis are the most used techniques (30% of the total number of techniques used in the sample); however, it is relevant that many of these papers used more than one statistical technique (Table 3).

RQ8 What EI assessment measures or models are applied in the analyzed documents?

The most widely used assessment measures are the WLEIS scale (20%), the MSCEIT scale (15%), and the 5G scale (14%) (Table 4).

### 4.2 Results of the co-word analysis of the most cited studies

This subsection includes results from the co-word analysis using the complete period 1998–2022 responding the proposed research questions.

RQ9 Which are the main categories resulting from the co-word analysis and the authors publish in each one?

To organize the selected studies, we considered three main categories: EI, leadership, and work teams (Table 5). According to the co-word analysis, the majority of the articles (53 out of 104) fall into the EI category, accounting for 17% of the total words analyzed and 52% of the total number of articles. Leadership accounts for 8% of the total number of words analyzed and 26% of all the articles reviewed (28 out of 104). The team category represents approximately 2% of the total number of words analyzed and 5% of the total number of articles.

RQ10 Which themes are studied in the subcategories resulting from the co-word analysis and the authors publish in each one?

The detailed study of co-word resulted in subcategories of EI, leadership, and work teams is included in Table 7. Lopez-Zafra et al. [105] and Villanueva and Sanchez [106] also have emotional intelligence and leadership. Meanwhile, Ahmed et al. [107], Harrison et al. [108], Hur et al. [109], Potter et al. [110], and Schlechter and Strauss [111] concur with leadership and team. Finally, Aritzeta et al. [112], Balamohan et al. [113], and Flores et al. [114] have all three subcategories in common. Côté et al. [115], Mandell and Pherwani [116], and Judge et al. [60] only have leadership subcategories while Stubbs Koman and Wolff [117] have team subcategories (Table 6).

### 4.3 Research objectives and findings of the most cited publications

RQ11 Which research objectives are studied in the analyzed articles and their correspondent authors?

Some articles have multiple objectives, so we catalogued the articles based on their primary or most relevant objective (Table 7).

One of the main contributions of this study is the classification of categories based on the research objectives of the analyzed articles in chronological order. The analysis is described in the following third-level sections.

#### 4.3.1 Leadership types: transformational, emergence, virtual, effective, healthy, resonant, and dissonant

Dulewicz and Higgs [118] establish a positive relationship between emotional intelligence and leadership. Judge and Bono [119] conclude that relationships exist between traits of the Big Five personality model (neuroticism, extraversion, openness to experience, and agreeableness) and transformational leadership behavior. However, Shao and Webber [120] point out that the Big Five model does not appear to be as effective a predictor of transformational leadership behaviors in China as compared to samples from Judge and Bono's [119] previous studies conducted in the United States. Later, Mandell and Pherwani [116] studied managers in different industries and concluded that there is a significant relationship between EI and transformational leadership style. Scott-Halsell et al. [121] found that transformational leaders use their EI skills to employ effective leadership skills, such as good interpersonal communication and team collaboration.

Ahmed et al. [107], Polychroniou [122], Prochazka et al. [123], and Schlechter and Strauss [111] support the idea that transformational leadership mediates the relationship between EI, effectiveness, performance, and team commitment. In other results, Hur et al. [109] and Mandell and Pherwani [116] found no significant gender differences in this relationship.

Ramsey et al. [124] pointed out that leaders with high levels of cultural intelligence also exhibit high levels of transformational leadership because they can understand their own behavior. Similarly, transformational leadership increases team motivation, performance, and effectiveness-related satisfaction [125]. Furthermore, Potter et al. [110] have discovered significant positive relationships between the EI of a group of project managers working in the construction industry and the likelihood of them adopting a transformational leadership style. Doan et al. [126] later confirmed the influence of EI, the mediating role of transformational leadership, and the moderating effect of organizational commitment on the relationship between EI and project success in company leaders from various areas. Along these lines, Mysirlaki and Paraskeva [127] provide evidence that transformational leadership moderates the relationship between leaders’ EI and effectiveness in a virtual team.

In regard to effective and transformational leadership, based on the Big Five model of personality factors, Lim Leung and Bozionelos [128] concluded that extroversion was the trait most associated with the prototypical notion of an effective leader and, in turn, linked to transformational leadership traits. The results also suggest that men and women may differ in the criteria they use to evaluate leaders.

Regarding effective leadership in the context of students in business administration programs, Offerman et al. [129] found a positive relationship between emotional competence and the attitudes of team members, as well as the leader's effectiveness. Rosete and Ciarrochi [54] argue that managers with higher levels of EI tend to achieve better business results; however, the small sample size limits the conclusion. The latter authors also suggest that, in terms of managing employee performance, it is equally important for an executive to know what task to manage in order to achieve good business results as it is to know how to effectively address his or her employees. Riggio and Reichard [130] subsequently found that emotional and social skills are essential for effective leadership.

In contrast, Weinberger [131] analyzed employees of a manufacturing company and concluded that there was no relationship between a leader's EI and his or her effectiveness, which contradicts the findings of Goleman [2,38]. Weinberger [131] points out the redundant importance given to the relationship between EI and leadership style and effectiveness in the literature. He also indicates that such relationships do not exist and are not necessary to further understand a leader's effectiveness in an organization. Finally, he claims that the ability-based definition of EI is of little use from an organizational perspective and suggests that definitions of EI should be broader and personality-based.

The literature review by Walter et al. [132] notes that although there are controversies between the definition of EI and its measurement, empirical research on EI and effective leadership has found that EI is a crucial element for the success of leadership development projects. Similarly, Boyatzis et al. [133] studied lead engineers at a multinational manufacturing company and found that social and emotional intelligence (SEI) competencies for effectiveness significantly predict the explanatory power of SEI over general mental ability and personality.

Edelman and van Knippenberg [134] subsequently provided evidence on the mediating role of EI on leadership effectiveness, controlling for cognitive ability and the Big Five personality traits. Cavaness et al. [135] point out that understanding the importance of EI and its connection to personality dimensions provides an additional tool for leaders to be more effective and successful.

In regard to emergent leadership, Wolff et al. [136] maintain that the behavioral abilities of group task coordination and the support and development of others would predict an individual being selected as an emergent team leader. Judge et al. [60]conclude that factors of the Big Five personality model, such as neuroticism, extraversion, openness to experiential appreciation, and conscientiousness, are the most consistent constructs of leadership across study settings and emergent and effective leadership. Judge et al. [61] later studied the relationship between intelligence and leadership and established that the degree of correlation between intelligence and leadership is considerably less than previously suggested in the literature.

In particular, Côté et al. [115] confirmed that EI is positively related to emergent leadership in small groups and point out the importance of understanding emotions according to the applied contexts. They also argue that the literature on studies that control for cognitive intelligence and personality traits is rather limited. Emery [137] concluded that emotional competencies play a complementary role in emergent leadership while, the same year, Walter et al. [138] provided an innovative view regarding the connection between emotion recognition and emergent leaders. The results show that task coordination behavior is a key mediating mechanism that transfers positively into the relationship of emotion recognition and extraversion as related to task coordination.

Among the few studies that analyze emergent leadership and motivation, Hong et al. [139] examined the role of EI and motivation to lead in predicting emergent leadership. The results support the idea that motivation to lead is essential for the emergence of leaders in different environments.

In another study, Cummings et al. [140] concluded that resonant leadership moderates the impact on nurses in the case of hospital restructuring. This means that nurses working under resonant leadership have less emotional exhaustion, less somatization, and better teamwork than nurses working for dissonant leaders.

#### 4.3.2 Leadership and emotion

In regard to the topic of leadership and emotions, Kelly and Barsade's [141] literature review shows that group emotion is the result of the combination of the affective composition of the individuals in a group and the affective context of the group itself. The authors proposed an organizational model to understand these affective influences and their impacts on group life. Pirola-Merlo et al. [142] analyzed how leaders influence the impact of affective events on team climate and performance. The findings show that potential obstacles within the organization have a negative impact on the team's work climate, although said effects can be neutralized by leaders adopting more facilitating and transforming styles.

That same year, Kellet et al. [143] established two behavioral pathways related to how an individual perceives leadership. One pathway is based on mental skills, such as task accomplishment, while the other pathway is based on emotional skills, such as empathy. Likewise, Rubin et al. [87] analyzed how leaders’ ability to recognize emotions and personality characteristics influences transformational leadership behavior performance.

#### 4.3.3 Leadership and team conflicts, abusive-subversive leaders, and pressure-support

Ayoko et al. [144] argue that teams with less-well-defined EI environments are associated with increased task and relationship conflict as well as conflict intensity. Clarke [145] later found that emotional competence and empathy measures explain additional variance in teamwork project managers’ competencies and conflict management.

Gavin et al. [146] showed that members of an organization who experience subversive leadership are less likely to trust their leaders, to feel satisfied with their jobs, and to stay at the organization. Moreover, the level of EI mediates the level of job satisfaction for these members, but not their intention to leave the organization.

Regarding subversive leaders, Pradhan and Jena [147] found no positive relationship in the moderating role of EI between abusive supervision (an interpersonal stressor) and employees’ intention to quit. In contrast, Li et al. [148] concluded that leaders who are always supportive of employees before any pressure occurs have higher EI levels than leaders who prefer to provide support after pressure occurs.

In the case of luxury hotels, Jung and Yoon [149] analyzed emotional contagion among burned-out employees and collective commitment. These authors concluded that more emotionally contaminated employees have higher levels of burnout, and the higher the burnout, the lower the commitment to the group.

#### 4.3.4 Leadership and work environment, leadership and interpersonal communication with team members, organizational behavior in the workplace, and leader behavior and well-being in the workplace

Although many studies attribute a positive influence on job success and well-being to EI, Zeidner et al. [150] criticize this finding, claiming that there's not enough research to support this relationship.

Ashkanasy and Daus [151] maintain that the trends have not been adequately distinguished and that EI has inappropriately been characterized as a variant of social intelligence, highlighting the relevant role of emotion in organizational behavior.

Nonetheless, recent scientific advances in the study of emotions, specifically regarding the role that emotions play in organizational behavior, are the basis of EI research. Liu et al. [152] maintain that leaders' positive emotional states are positively related to employees’ positive communication behaviors toward their managers.

#### 4.3.5 Performance, leaders, teams, and work

Poon Teng Fatt [76] concluded that the EI competencies of a team's members effectively influence the team and its performance. Likewise, Offermann et al. [129] maintain that emotional capabilities assessed by a competency model influence both individual and team performance. Lopes et al. [41] later related EI to several job performance indicators, although the sample size limits their results. Stubbs Koman and Wolff [117] also confirm a positive relationship between team leaders' EI competencies and their team's performance. According to Chang et al. [153], both team members' average EI and emotionally intelligent leadership have a positive influence on internal confidence and team performance. These authors recommend further research to be conducted on EI and emotional management at the group level.

Considering different industries, Stein et al. [154] concluded that executives with higher levels of self-esteem and better problem management skills are more likely to create companies with higher profits. It is also vital for managers to know how to intersperse social competencies with task-specific competencies in order to achieve successful work performance.

Zhang and Fan [155] confirmed that international participation and contract type moderate the relationships between certain EI factors and project performance. These authors also confirmed that team members with high emotional competence generally enhance team success and show superior personal performance at work, which is supported by other authors [118,156]. Neil et al. [157] subsequently provided insights into the effective use of EI and leaders’ attitudes to benefit team cohesion and performance.

Vijayabanu and Arunkumar [158] analyzed personality traits and team performance and verified the relationship between personality traits and emotions affecting team performance. Similarly, Bartone et al. [159] assessed the influence of psychological toughness, social judgment, and the Big Five personality dimensions on leader performance at a military academy. The Big Five, extroversion, toughness, and a tendency toward social judgment predict this performance.

#### 4.3.6 Other objectives

Carmeli [15] corroborates the importance of EI within the context of senior management in the public sector, as it increases positive work attitudes and task performance. A year later, Law et al. [86] analyzed several evaluations including supervisors' assessments of their employees' task performance, and the employees' assessments of their supervisors’ EI and job performance, as well as the performance of their peers. The results showed that peer ratings were significant predictors of job performance ratings as provided by supervisors after controlling for the Big Five personality dimensions. Law et al. [86] argue that the concept of EI differs from the concept of personality.

On another note, Espinosa et al. [160] studied project success, which was determined by evaluating the impact of emotional leadership competencies, intellectual intelligence, and management intelligence on project success according to varying degrees of complexity. They found that trust complexity moderates the relationship between EQ and project success. They investigated the moderating effect of project complexity on the relationship between project managers’ leadership competencies and their project success. The results show that EQ and MQ are correlated with project success but are moderated differently depending on the complexity.

In recent years, authors such as Fareed et al. [161], Zhang and Hao [162], and Zhang et al. [163] have tackled this issue. More specifically, Fareed et al. [161] point out that EI, project managers' intellectual competencies, and transformational leadership contribute significantly to project success. Zhang and Hao [162] have determined that a project manager's EI influences the willingness to achieve objectives in a project; that is, it has a more effective influence on leading team members (mediating team cohesion and duration in project management). Zhang et al. [163] investigated the factors influencing the relationship between project managers' EI and project performance, concluding that project managers' EI positively and significantly influences project performance.

Finally, within the context of employee innovation, Jena and Goyal [164] studied the mediating effect of person-group fit and adaptive performance on employee innovation.

## 5 Conclusions

This review of the selected articles indicates that the diversity of conceptualizations of EI can lead to contradictory concepts and may impair one's understanding of the construct [[47], [48], [49]]. This diversity of concepts is reflected in different measurement scales, thus raising the question of the comparability of EI measures.

Authors such as Conté [51] and Côté et al. [115] debate about the best way to measure IE. Conté [51] points out that only the ability test scores showed incremental validity over the Big Five personality traits and gender. Rosete and Ciarrochi [54] remark that the overall EI score did not correlate with any of the 16 personality factors. These results support previous research that showed that MSCEIT scores are distinguishable from personality [52]. Similarly, authors such as Brackett & Mayer [52], Matthews et al. [53], and Mayer et al. [28] found low correlations between the constructs of the MSCEIT model and the EQ-i model. Chandrapal et al. [165] argue that there could be bias in self-report tests or a limitation in their assessments.

Academics who are advocates of EI measurement accept that the different measures have adequate internal consistency reliability, both in self-reports and competence-based measures [2,29,30]. Dulewicz and Higgs [118] concluded that the construct of emotional intelligence presents validity and reliability measured through a questionnaire derived from a competence-based measure, which backs up Goleman's research [2]. Similarly, Stein et al. [154] support using the EQ-i as a functional tool in an individual's assessment and development in executive roles.

From the performance analysis of the selected documents, several conclusions are drawn. The period being studied ran from 1998 to 2022, with growing interest in the field in recent years, specifically 2017, 2018, and 2022. The countries samples more frequent are the United States and the United Kingdom. Moreover, the most widely studied industries are higher education, healthcare and hospitals, and construction.

Schutte et al. [32] and Judge et al. [60] are the most cited authors in WoS while Carmeli [15] is the most cited author in Scopus. The reviewed articles were published in 73 different scientific journals, with “Leadership and Organization Development Journal” at the top of the ranking. The top thirteen journals included with the most published papers are classified into the categories: Management (11); Applied Psychology (4); Business (2); Multidisciplinary (2); Industrial Relations & Labor (1); Nursing (1); Psychology (1); and Social Psychology (1). The most frequent category among them is Management, followed by Applied Psychology. In terms of ranking of these top journals, the most frequent ranking of these top journals belonging to Journal Citation Report-Social Sciences (JCR-SCCI) are quartile Q1 (Nursing, Business, Industrial Relations & Labor, Psychology, and Multidisciplinary) followed by Q3 (Management, Social Psychology).

Most of the studies were empirical (79%), while others were primarily conceptual (21%). The most frequently used techniques were correlation and regression analyses; however, many quantitative studies applied more than one technique. Additionally, the most frequently applied measures of emotional intelligence are the Wong-Law Emotional Intelligence Scale (20%), the Mayer-Salovey-Caruso Emotional Intelligence Test (15%), and the Five-Item General Leadership Impression Scale (14%).

The results of co-word analysis led to the conclusion that the reviewed publications mainly focus on the study of emotional intelligence followed by leadership in the section on categories.

Following is a summary of the main conclusions of the most cited academics’ research goals in the three main categories. As far as transformational leadership, conclusions support the positive influence of EI and the mediating role of transformational leadership, as well as the moderating effect of organizational commitment on the relationship between EI, performance, and project success [118,126].

The traits of the Big Five personality model have a positive correlation with transformational leadership behavior [119]; however, the results depend on the country where the sample is collected, as the predictor may be less significant in certain countries [120]. Some authors confirm a significant positive relationship between transformational leadership style and emotional intelligence [116]. Furthermore, Lim Leung and Bozionelos [128] analyzed transformational leadership and efficacy by applying the Big Five model of personality factors, concluding that extroversion is the trait most highly associated with effective leaders and that efficacy, in turn, is linked to transformational leadership traits.

Regarding effective leadership, the literature supports the positive relationship between emotional competence and team members' attitudes, as well as the leader's effectiveness [129]. Other authors argue that managers with higher levels of EI tend to achieve better business results. Specifically, in terms of managing employee performance, it is equally important for an executive to know what task to manage in order to achieve good business results and to effectively address their employees [54]. Emotional and social skills are also vital for effective leadership [130]. In contrast, Weinberger [131] concludes that there is no relationship between leadership effectiveness and EI.

As mentioned earlier, controversies arise between defining and measuring EI. Thus, empirical research on EI and effective leadership has established EI as crucial element for the success of leadership development projects [132]. Furthermore, there is evidence that EI mediates leadership effectiveness by controlling for cognitive ability and the Big Five personality traits [134]. Understanding the importance of EI and its connection to personality dimensions therefore provides an additional tool to make leaders more effective and successful [135].

An emerging team leader is selected primarily on the basis of behavioral skills of coordinating group tasks and supporting others [136]. Factors of the Big Five personality model are therefore the most consistent constructs of leadership across study settings and emergent and effective leadership [60]. Consequently, there is a positive correlation between EI and emergent leadership and it is important to understand emotions depending on their applied contexts [115].

Considering leadership and emotion, group emotion is the result of a combination of the affective composition of the individuals in the group and the affective context of the group itself [141]. Consequently, obstacles can have a negative impact on the team's work climate, although this impact can be neutralized by leaders adopting more facilitating and transforming styles.

Based on the authors we reviewed, in terms of performance based on a competency model, emotional capabilities were found to influence individual and/or team performance [41,77,87,88,118,130,142,152].

Conclusions regarding leadership and team conflicts indicate that measures of emotional intelligence and empathy explain additional variance in teamwork, project managers’ competencies, and conflict management [145]. Similarly, Ayoko et al. [144] argue that teams with less-well-defined emotional intelligence environments are associated with increased task and relationship conflict and conflict intensity. Moreover, more emotionally contaminated employees have higher levels of burnout, and the higher the burnout, the lower the commitment to the group [149].

Although there is a great deal of research attributing work success and well-being to the positive impact of EI, there is little research that supports such a relationship between leadership and the work environment [150].

It is important to address the limitations of the selected documents. Academics such as Araujo and Taylor [156] and Hur et al. [109] have established that ability measures tend to be less biased than self-report measures. The results of some studies are limited as they are based on a simulation environment using a sample of students [123]. Additionally, the use of small sample sizes [41,54,60,145,156,166,167] and data collected from convenience samples [122] make it impossible to generalize the results. Accordingly, a potential line of future research would be to analyze EI in different cultures and across different sectors [15]. Further recommendations include conducting more EI assessments based on ability measures, different models, and using mixed assessments [129].

## 6 Future research

The use of mindfulness is being developed on both a personal and job level as empirical evidence shows that it has a positive influence on people regulating their emotions and behaviors. Consequently, it has a positive influence on well-being, work performance, and interpersonal relationships, in addition to many other benefits. Likewise, mindfulness practice is recommended for leaders as it helps develop emotional intelligence, social skills and support within organizations [[97], [98], [99]]. The practice of mindfulness therefore favors the development of resilience and counteracts the effects of BANI and VUCA environments [18,95].

Intergenerational studies (Baby Boomers, Generation X, Generation Y, and Generation Z) continue to be a current topic, as there are papers from 2023 that discuss this topic but they have not been included in the period under study [168].

Artificial intelligence is among the major advances in technology that affect companies, improving service quality and customer satisfaction. Specifically, worthy of mention are machine learning models that generate content similar to how the human brain learns and responds to data [90,102,103]. However, although artificial intelligence can go beyond human abilities to solve complex problems and can generally be useful to improve the internal efficiency of companies, it is still very limited in terms of managing the emotions of employees within an organization [18].

Finally, we suggest prospective studies using different scales to measure the EI of leaders and individuals within the same organization in order to compare findings and generalize conclusions. The subject invites further research on the topic, jointly assessing the three topics reviewed, the emotional intelligence of leaders and the work team in the same organizational context. The findings could lead to an improved selection process for leaders and team members within an organization.

## Acknowledgement of funding sources

The 10.13039/100009473University of Malaga (Spain) funded the publication fees.

## Author contribution statement

All authors listed have significantly contributed to the development and the writing of this article.

## Data availability statement

An asterisk (*) marks the 104 reviewed documents in the Reference Section.

## Additional information

No additional information is available for this paper.

## Declaration of competing interest

The authors declare that they have no known competing financial interests or personal relationships that could have appeared to influence the work reported in this paper.

## Figures and Tables

**Table 1:** Country sample and activity sectors.

**Country sample**	**Percentage**	**Activity sector**	**Percentage**
United States	23.6%	Training (MBA, students, university)	21.7%
United Kingdom	14.6%	Hospital/Health	16.7%
China	12.4%	Construction	11.7%
Australia	7.9%	Public and private organization	10.0%
Canada	4.6%	Manufacturing	10.0%
Spain	4.6%	Technology and Information	10.0%
South Korea	3.5%	Banking and Finance	5.0%
India	3.5%	Different sectors and industries	5.0%
New Zealand	2.3%	Organizations in various industries/general	5.0%
Pakistan	2.3%	Professional Services	5.0%
Saudi Arabia	2.3%	Military	5.0%
Vietnam	2.3%	Total	100%
**Multi-countries samples**			
Ten papers included a multi-country sample (more than two countries)	19.2%		
One paper includes 13 countries ^(1)^	1.2%		

Note. Percentages do not add up to 100%. (1) Brazil, Czech Republic, Denmark, Greece, Israel, Japan, Netherlands, Peru, Serbia, Singapore, South Africa, and Sweden. Each classification adds up to 100%. Source: Own elaboration.

**Table 2:** Most cited authors and journals of the reviewed articles, ranking and categories.

Most cited authors			Journals of the reviewed articles, ranking and categories			
Authors	WoS	Scopus	Scientific journal	Ranking 2022: Categories	Nº	%
Schutte et al. [32]	1796	1796	Leadersh. Organ. Dev. J.	JCR-Q3 Management – SSCI	10	9.62
Judge et al. [60]	1396	1616	J. Manage. Psychol.	JCR-Q3 Management – SSCI; JCR-Q2 Psychology, Applied - SSCI	5	4.81
Judge and Bono [119]	644	745	Leadersh. Q.	JCR-Q1 Psychology – SSCI; JCR-Q1 Multidisciplinary –SSCI	5	4.81
Law et al. [86]	642	695	Front. Psychol.	JCR-Q1 Psychology – SSCI; JCR-Q1 Multidisciplinary - SSCI	4	3.85
Kelly and Barsade [141]	596	680	J. Appl. Psychol.	JCR-Q1 Applied Psychology – SSCI; JCR-Q1 Management - SSCI	4	3.85
Rubin et al. [87]	350	351	J. Organ. Behav.	JCR-Q1 Business – SSCI; JCR-Q1 Applied Psychology – SSCI	3	2.88
Zeidner et al. [150]	335	335		JCR-Q1 Management - SSCI		
Rosete and Ciarrochi [54]	299	301	Team Perform. Manag.	JCR-Q3 Management - ESCI	3	2.88
Judge et al. [61]	296	341	Acad. Manag. Ann.	JCR-Q1 Business – SSCI; JCR-Q1 Management - SSCI	2	1.92
Ashkanasy and Daus [151]	284	307	Empl. Relat.	JCR-Q2 Industrial Relations & Labor – SSCI	2	1.92
Pirola-Merlo et al. [142]	254	283		JCR-Q4 Management - SSCI		
Mandell and Pherwani [116]	238	238	Int. J. Organ. Anal.	JCR-Q3Management- ESCI	2	1.92
Wolff et al. [136]	236	236	Int. J. Project Management	JCR-Q1Management - SSCI	2	1.92
Lopes et al. [41]	185	212	J. Nurs. Manag.	JCR-Q2 Management – SSCI; JCR-Q1 Nursing - SSCI	2	1.92
Kellet et al. [143]	146	166	Small Group Res.	JCR-Q4 Applied Psychology – SSCI	2	1.92
Riggio and Reichard [130]	139	167		JCR-Q3 Management – SSCI		
Boyatzis [39]	132	7		JCR-Q3 Social Psychology - SSCI		
Côté et al. [115]	128	133	60 journals only, with one article in each journal.		60	0.96
Liu et al. [152]	125	120	*Total*		*104*	*100%*
Cummings et al. [140]	120	120				
Clarke [145]	119	141				
Walter et al. [132]	113	131				
Stubbs Koman and Wolff [117]	112	126				
Ayoko et al. [144]	94	107				
Hur et al. [109]	86	91				
Offermann et al. [129]	83	83				
Carmeli [15]	–	407				
Dulewicz and Higgs [118]	–	97				

Note: Social Sciences Citation Index (SSCI) and Emerging Sources Citation Index (ESCI). See the reference section, where an asterisk (*) marks the 104 reviewed articles. Note. Nº = Number of articles; % % e-share. Source: Own elaboration.

**Table 3:** Design Research of the documents and techniques of empirical research.

Design Research		Authors	Num.	Perc.	Techniques	
**Conceptual**		Alferaih [169]; Ashkanasy and Daus [151]; Balamohan et al. [113]; Bellack and Dickow [170]; Bencsik and Bognár [171]; Boyatzis [39]; Riggio and Reichard [130]; Cavaness et al. [135]; Edgar et al. [172]; Flores et al. [114]; Hopkins et al. [173]; Ireland [174]; Kelly and Barsade [141]; Lambert [175]; McCallin and Bamford [176]; McCleskey [25]; Scott-Halsell et al. [121]; Walter et al. [132]; Ward [177]; Yusof et al. [178]; Zhang et al. [163]; Zeidner et al. [150]	22	21,2		
					**Applied to qualitative**	**Num.**
**Empirical**	**Qualitative**	Davies et al. [179]; Mansel and Einion [180]			Interview	2
					**Applied to Quantitative**	**Num.**
**Empirical**	**Quantitative**	Ahmed et al. [107]; Alotaibi et al. [181]; Araujo and Taylor [156]; Aritzeta et al. [112]; Arnatt and Beyerlein [166]; Ayoko et al. [144]; Bartone et al. [159]; Boyatzis et al. [133]; Carmeli [15]; Chandrapal et al. [165]; Chang et al. [153]; Chaudhary et al. [182]; Clarke [145,167]; Côté et al. [115]; Cummings et al. [140]; Doan et al. [126]; Downey et al. [183]; Du et al. [184]; Dulewicz and Higgs [118]; Edelman and van Knippenberg [134]; Emery [137]; Espinosa et al. [160]; Fareed et al. [161]; Furukawa and Kashiwagi [185]; Gavin et al. [146]; Harrison et al. [108]; Higgs and Aitken [186]; Hong et al. [139]; Hur et al. [109]; Jena and Goyal [164]; Judge and Bono [119]; Judge et al. [60]; Judge et al. [61]; Jung and Yoon [149]; Kellet et al. [143]; Stubbs Koman and Wolff [117]; Langford et al. [187]; Law et al. [86]; Li et al. [148]; Lim Leung and Bozionelos [128]; Liu et al. [188]; Liu et al. [152]; Lopes et al. [41]; Lopez-Zafra et al. [105]; Mandell and Pherwani [116]; McCormack and Mellor [69]; McKeown and Bates [189]; Mindeguia et al. [190]; Mysirlaki and Paraskeva [127]; Neil et al. [157]; Offermann et al. [129]; Ozcelik et al. [191]; Pirola-Merlo et al. [142]; Polychroniou [122]; Poon Teng Fatt [76]; Potter et al. [110]; Pradhan and Jena [147]; Prochazka et al. [123]; Pryke et al. [192]; Ramsey et al. [124]; Rezvani et al. [193,194]; Rosete and Ciarrochi [54]; Rubin et al. [87]; Schlechter and Strauss [111]; Schutte et al. [32]; Shao and Webber [120]; Stein et al. [154]; Vijayabanu and Arunkumar [158]; Villanueva and Sánchez [106]; Walter et al. [138]; Wei et al. [195]; Weinberger [131]; Wilderom et al. [196]; Wittmer and Hopkins [197]; Wolff et al. [136]; Zhang and Fan [155]; Zhang and Hao [162]; Zhang et al. [125]	82	78,9	Correlation	46
					Regression analysis	43
					Descriptive statistics	21
					Factor analysis	13
					ANOVA	10
					Structural equation modeling	8
					Hierarchical regression	5
					Cluster analysis	2
					Partial Least Square (PLS)	3
					T-student test	1
					Covariance analysis	1
					Inequality analysis	1
	
	
						156
		Total	104	100		156

Note. Nº, Number of documents o number of times the techniques have been used; Perc., Percentage. Authors are in alphabetical order in the table. Source: Own elaboration.

**Table 4:** Methods of **assessing** emotional intelligence.

Methods	Times used
Wong-Law Emotional Intelligence Scale (WLEIS)	16
Mayer-Salovey-Caruso EI Test (MSCEIT)	9
Five-item General Leadership Impression Scale	8
Bar-On Emotional Quotient Inventory (EQ-i)	7
Schutte Self Report Inventory (SSRI)	6
Emotional Competence Inventory (ECI)	4
Trait Meta Mood Scale (TMMS)	4
Workgroup Emotional Intelligence Profile (WEIP)	3
Dulewicz and Higgs Test	2

**Table 5:** Classification of documents by main categories.

Categories	Authors	Categories	Authors	Categories	Authors
Emotional intelligence	Ahmed et al. [107]; Alferaih [169]; Alotaibi et al. [181]; Araujo and Taylor [156]; Aritzeta et al. [112]; Ayoko et al. [144]; Balamohan et al. [113]; Bartone et al. [159]; Bellack and Dickow [170]; Bencsik and Bognár [171]; Boyatzis [39]; Boyatzis et al. [133]; Cavaness et al. [135]; Chandrapal et al. [165]; Chang et al. [153]; Clarke [145,167]; Côté et al. [115]; Davies et al. [179]; Doan et al. [126]; Downey et al. [183]; Edelman and van Knippenberg [134]; Edgar et al. [172]; Emery [137]; Espinosa et al. [160]; Furukawa and Kashiwagi [185]; Harrison et al. [108]; Hong et al. [139]; Hopkins et al. [173]; Hur et al. [109]; Jena and Goyal [164]; Li et al. [148]; Mandell and Pherwani [116]; Mansel and Einion [180]; McCallin and Bamford [176]; McCleskey [25]; Mysirlaki and Paraskeva [127]; Neil et al. [157]; Polychroniou [122]; Potter et al. [110]; Pradhan and Jena [147]; Riggio and Reichard [130]; Scott-Halsell et al. [121]; Stein et al. [154]; Stubbs Koman and Wolff [117]; Vijayabanu and Arunkumar [158]; Walter et al. [138]; Weinberger [131]; Wilderom et al. [196]; Wittmer and Hopkins [197]; Zhang and Fan [155]; Zhang and Hao [162]; Zhang et al. [125]; Zhang et al. [163]	Leadership	Araujo and Taylor [156]; Aritzeta et al. [112]; Arnatt and Beyerlein [166]; Balamohan et al. [113]; Bartone et al. [159]; Bellack and Dickow [170]; Chandrapal et al. [165]; Chang et al. [153]; Clarke [145]; Dulewicz and Higgs [118]; Edelman and van Knippenberg [134]; Edgar et al. [172]; Espinosa et al. [160]; Flores et al. [114]; Furukawa and Kashiwagi [185]; Hong et al. [139]; Hopkins et al. [173]; Lambert [175]; Langford et al. [187]; Liu et al. [152]; McCleskey [25]; McKeown and Bates [189]; Mindeguia et al. [190]; Neil et al. [157]; Ozcelik et al. [191]; Potter et al. [110]; Rosete and Ciarrochi [54]; Scott-Halsell et al. [121]	Team	Aritzeta et al. [112]; Ayoko et al. [144]; Balamohan et al. [113]; Flores et al. [114]; Schlechter and Strauss [111]

Note: Authors are in alphabetical order in table. An asterisk (*) marks the 104 reviewed documents in the Reference Section. Source: Own elaboration and Atlas.ti.

**Table 6:** Subcategories: emotional intelligence, leadership, and team.

Subcategories emotional intelligence		Subcategories leadership		Subcategories Team	
Group emotional intelligence	Lopez-Zafra et al. [105]	Effective leadership	Edelman and van Knippenberg [134]; Harrison et al. [108]; Weinberger [131]; Yusof et al. [178]	Team leadership	Harrison et al. [108]
Trait emotional intelligence	Villanueva and Sánchez [106]	Emotion Leadership	Côté et al. [115]	Interdisciplinary teamwork	McCallin and Bamford [176]
Team emotional intelligence	Du et al. [184]; Mindeguia et al. [190]; Rezvani et al. [193]; Wei et al. [195]	Emotional intelligent leadership	McKeown and Bates [189]	Intrateam trust	Chang et al. [153]
		Leadership competency	Harrison et al. [108]; Zhang et al. [163]	Interdisciplinary teamwork	McCallin and Bamford [176]
		Leadership development	Riggio and Reichard [130]	Team climate	Ayoko et al. [144]
		Leadership emergence	Côté et al. [115]; Emery [137]	Team commitment	Schlechter and Strauss [111]
		Leadership failure	Bellack and Dickow [170]	Team diversity	McCallin and Bamford [176]
		Leadership impact	Harrison et al. [108]	Team effectiveness	Ahmed et al. [107]; Hur et al. [109]; Zhang and Hao [162]
		Leadership self-efficacy	Villanueva and Sánchez [106]	Team leaders	Stubbs Koman and Wolff [117]
		Leadership styles	Ahmed et al. [107]; Bellack and Dickow [170]; Li et al. [148]; Mandell and Pherwani [116]; Zhang et al. [125]	Team learning	Bencsik and Bognár [171]
		Non-profit board chair leadership	Harrison et al. [108]		
		Nurse leadership	Mansel and Einion [180]		
		Personality and leadership	Judge et al. [60]		
		Relational leadership	Harrison et al. [108]		
		Team leadership	Harrison et al. [108]		
		Transformational leadership	Doan et al. [126]; Lopez-Zafra et al. [105]; Polychroniou [122]; Prochazka et al. [123]; Ramsey et al. [124]; Schlechter and Strauss [111]; Scott-Halsell et al. [121]		
		Transformational leadership style	Hur et al. [109]; Potter et al. [110]		

Note: Authors are in alphabetical order in table. An asterisk (*) marks the 104 reviewed documents in the Reference Section. Source: Own elaboration and Atlas.ti.

**Table 7:** Research objectives.

Research objective	Authors
Leadership (Transformational, emergence, virtual, effective, health, effectiveness)	Ahmed et al. [107]; Alotaibi et al. [181]; Aritzeta et al. [112]; Arnatt and Beyerlein [166]; Bellack and Dickow [170]; Boyatzis et al. [133]; Cavaness et al. [135]; Chandrapal et al. [165]; Chaudhary et al. [182]; Côté et al. [115]; Cummings et al. [140]; Dulewicz and Higgs [118]; Doan et al. [126]; Edelman and van Knippenberg [134]; Emery [137]; Harrison et al. [108]; Higgs and Aitken [186]; Hong et al. [139]; Hopkins et al. [173]; Hur et al. [109]; Ireland [174]; Judge and Bono [119]; Judge et al. [60]; Judge et al. [61]; Lambert [175]; Langford et al. [187]; Lim Leung and Bozionelos [128]; Lopez-Zafra et al. [105]; Mandell and Pherwani [116]; Mansel and Einion [180]; McCallin and Bamford [176]; McCormack and Mellor [69]; McKeown and Bates [189]; Mysirlaki and Paraskeva [127]; Offermann et al. [129]; Polychroniou [122]; Potter et al. [110]; Prochazka et al. [123]; Ramsey et al. [124]; Riggio and Reichard [130]; Rosete and Ciarrochi [54]; Schlechter and Strauss [111]; Scott-Halsell et al. [121]; Shao and Webber [120]; Walter et al. [132]; Ward [177]; Weinberger [131]; Wittmer and Hopkins [197]; Wolff et al. [136]; Zhang et al. [125]
Leadership and emotion	Kellet et al. [143]; Kelly and Barsade [141]; Mindeguia et al. [190]; Pirola-Merlo et al. [142]; Rubin et al. [87]; Walter et al. [138]; Yusof et al. [178]
Leadership and team conflicts/abusive or subversive leader/support and pressure	Ayoko et al. [144]; Clarke [145]; Flores et al. [114]; Gavin et al. [146]; Jung and Yoon [149]; Li et al. [148]; Pradhan and Jena [147]; Rezvani et al. [194]
Leadership and work environment/leadership and interpersonal communication with team members/Organizational behavior in the workplace and leader behavior/well-being in the workplace	Ashkanasy and Daus [151]; Balamohan et al. [113]; Edgar et al. [172]; Liu et al. [188]; Liu et al. [152]; McCleskey [25]; Pryke et al. [192]; Zeidner et al. [150]
Performance (leader, team, work)	Araujo and Taylor [156]; Alferaih [169]; Bartone et al. [159]; Chang et al. [153]; Lopes et al. [41]; Neil et al. [157]; Offermann et al. [129]; Ozcelik et al. [191]; Poon Teng Fatt [76]; Rezvani et al. [193]; Stein et al. [154]; Stubbs Koman and Wolff [117]; Vijayabanu and Arunkumar [158]; Villanueva and Sánchez [106]; Wei et al. [195]; Wilderom et al. [196]; Zhang and Fan [155]
Other objectives (Organizational and emotional intelligence training of the leadership and teamwork/Skills in the workplace/Team creativity/Employee innovation/Leadership, project success, and project management/Management success)	Bencsik and Bognár [171]; Carmeli [15]; Clarke [167]; Du et al. [184]; Espinosa et al. [160]; Fareed et al. [161]; Furukawa and Kashiwagi [185]; Jena and Goyal [164]; Law et al. [86]; McCallin and Bamford [176]; Scott-Halsell et al. [121]; Zhang and Hao [162]; Zhang et al. [163]

Note: Authors are in alphabetical order in table. An asterisk (*) marks the 104 reviewed documents in the Reference Section. Source: Own elaboration and Atlas.ti.
